# Dual-scope–assisted endoloop resection of a giant esophageal fibrovascular polyp

**DOI:** 10.1016/j.vgie.2025.12.003

**Published:** 2025-12-26

**Authors:** Romario Ruiz, Paulo Bardalez-Cruz, Bryan Medina-Morales, Jairo Asencios-Cusihuallpa, Luis Marin-Calderón, Carlos Pachas, Harold Benites-Goñi

**Affiliations:** 1 Departamento de Aparato Digestivo, Hospital Nacional Edgardo Rebagliati Martins, Lima, Peru; 2 Departamento de Anatomía Patológica, Hospital Nacional Edgardo Rebagliati Martins, Lima, Peru; 3 Vicerrectorado de Investigación, Universidad San Ignacio de Loyola, Lima, Peru

## Abstract

**Background and Aims:**

Giant fibrovascular polyps of the esophagus are rare benign tumors. Although open surgery has been the standard treatment, it carries significant morbidity. Endoscopic resection is a promising alternative, but experience with large lesions remains limited. We report a case illustrating its feasibility and safety.

**Methods:**

An 86-year-old man with comorbidities presented with progressive dysphagia and oral protrusion of a soft mass. Endoscopy revealed a 10-cm pedunculated polyp distal to the cricopharyngeus. CT showed no invasion or lymphadenopathy. Resection was performed with the patient under general anesthesia using a dual endoscope technique to place an endoloop, followed by hot snare excision and thermocoagulation.

**Results:**

The resection was completed successfully without intraoperative adverse events. After the procedure, the patient experienced aspiration-related pneumonia, which was managed with intravenous antibiotics. The patient was discharged in good general condition. No recurrence was observed during 2 years of clinical and endoscopic follow-up.

**Conclusions:**

Endoscopic resection is a safe and effective minimally invasive option for giant esophageal polyps, even in high-risk patients, and may be considered as a first-line treatment.

## Introduction

Fibrovascular polyps of the esophagus are rare benign tumors composed of fibrous, vascular, and adipose tissue. They typically originate in the cervical esophagus, just distal to the cricopharyngeal muscle, and are usually intraluminal. They are considered giant when exceeding 5 cm in length and can reach considerable sizes without symptoms because of the distensibility of the esophageal wall.^1^ However, progressive growth may lead to dysphagia, oral regurgitation of the mass, weight loss, or even airway obstruction. Cases of sudden death from asphyxiation have been reported, making resection mandatory even in the absence of severe symptoms.^2^^,^^3^

Traditionally, open surgical resection has been the treatment of choice for large lesions. However, this approach is associated with higher morbidity, including esophageal fistulas, transient dysphonia, and prolonged hospitalization. In contrast, endoscopic resection has emerged as an effective and safer alternative, with lower risk of adverse events and recurrence.^4^^,^^5^ To our knowledge, published experience with endoscopic removal of giant polyps remains limited, which has hindered its adoption as a first-line option. The following case illustrates the feasibility and safety of the endoscopic approach for a giant lesion.

### Case presentation

**Figure 1:**
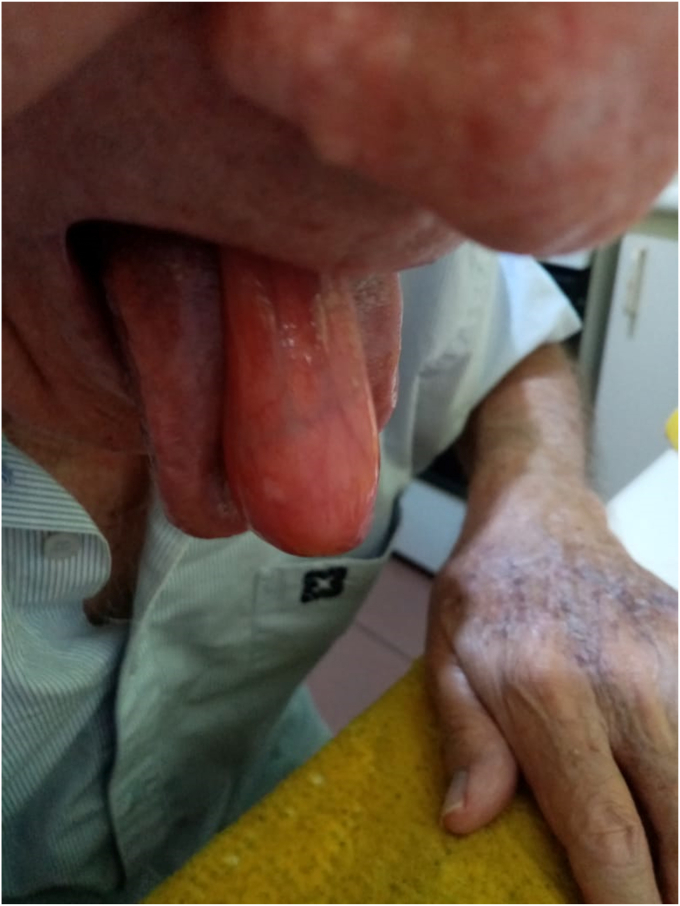
Esophageal fibrovascular tumor protruding through the oral cavity.

An 86-year-old male patient with Parkinson disease, trigeminal neuralgia, and bilateral hip arthroplasty was referred for progressive dysphagia, weight loss, and occasional oral protrusion of a soft, painless mass over 1 year (Fig. 1).

**Figure 2:**
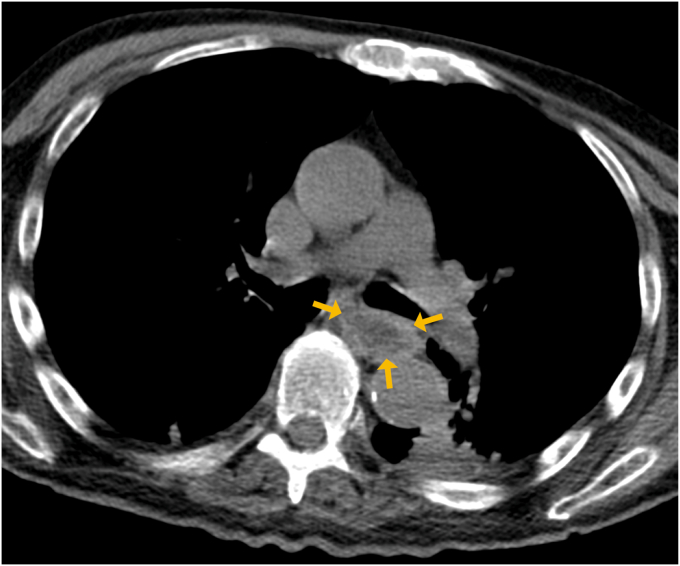
Axial contrast-enhanced CT scan showing a well-circumscribed hypodense intraluminal esophageal lesion (*yellow arrows*), with no evidence of wall invasion or involvement of adjacent structures.

Upper endoscopy revealed a giant pedunculated polypoid lesion arising just distal to the cricopharyngeus, measuring approximately 10 cm in length and 2 cm in diameter. The distal portion showed necrosis, and the midsegment had ulcerations, likely from chronic pedicle torsion. Contrast-enhanced CT confirmed an intraluminal, pedunculated mass without mural invasion or lymphadenopathy (Fig. 2).

The diagnosis was based on endoscopic and radiologic findings. Biopsy was avoided because superficial samples are often nondiagnostic and may increase the risk of bleeding.

**Figure 3:**
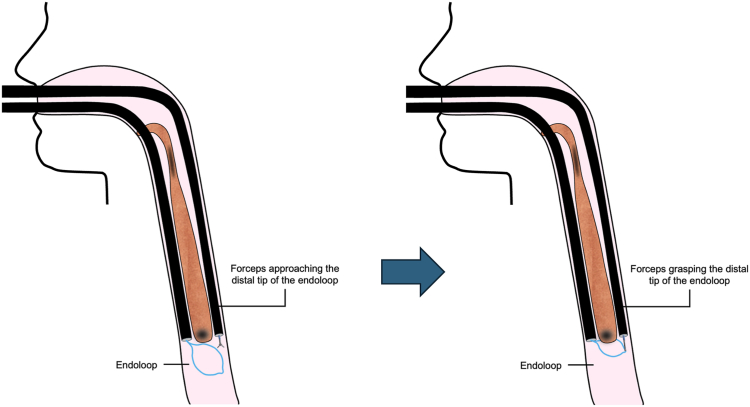
Schematic representation of the dual-scope–assisted endoloop technique. A pediatric endoscope is introduced alongside the main endoscope to grasp the distal tip of the endoloop with forceps, providing traction and stability for precise placement at the base of the polyp.

Given the lesion's size and cervical location, the procedure was performed with the patient under general anesthesia with orotracheal intubation. Using a conventional endoscope (EG-600ZW; Fujifilm, Tokyo, Japan), we made initial attempts to place an endoloop at the polyp base that were unsuccessful because of its size and mobility. To overcome this, a second pediatric endoscope was introduced, allowing traction and successful endoloop placement at the pedicle base (Fig. 3). The endoloop was positioned entirely within the esophageal lumen without exteriorizing the lesion, avoiding manipulation of a large, friable, and partially necrotic mass that could increase the risk of trauma, bleeding, or airway compromise.

**Figure 4:**
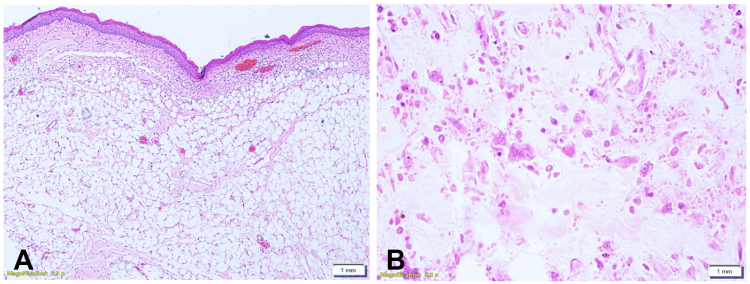
**A,** Hematoxylin and eosin staining demonstrating that the polyp was lined by benign squamous epithelium and composed of a mixture of mature adipose tissue and fibrous zones at lower magnification (orig. mag. ×40). **B,** At higher magnification, the lesion showed areas of necrosis. The base of the polyp was not involved (orig. mag. ×200).

Resection was carried out using a 25-mm hot Hexagonal snare (Micro-Tech, Nanjing, China), and the specimen was retrieved intact with a Roth Net retrieval device (Micro-Tech). A small residual base was intentionally left to reduce traction and perforation risk; this remnant was expected to undergo ischemic necrosis after endoloop placement. Hemostasis was achieved with targeted thermocoagulation, and final inspection confirmed a patent lumen with no bleeding or perforation (Video 1, available online at www.videogie.org). Histologic examination revealed a benign fibrovascular polyp composed of mature adipose and fibrous tissue, lined by squamous epithelium, with negative resection margins (Fig. 4).

Recovery was uneventful, and the patient was discharged on the fourth day in good condition. At 2-year follow-up, the patient remains asymptomatic with no evidence of recurrence.

## Conclusions

Endoscopic resection can be a safe, effective, and minimally invasive alternative for treating giant esophageal fibrovascular polyps, particularly in elderly or high-risk patients for whom surgery is unsuitable. Although endoscopic submucosal dissection (ESD) may be considered for some esophageal polyps, in this case, the lesion's origin immediately below the cricopharyngeus made ESD technically challenging because of limited visualization and an increased risk of perforation. The dual-scope–assisted endoloop technique provided stable traction and secure strangulation of the vascular stalk, ensuring controlled resection and hemostasis while minimizing manipulation of the cervical esophagus.

## Patient consent

Written informed consent for publication of this case and accompanying images was obtained from the patient’s son.

## Disclosure

All authors disclosed no financial relationships.

## Supplementary data

Video 1 Dual-scope–assisted endoloop resection of a giant esophageal fibrovascular polyp: step by step.
